# Abnormal expansion of naïve B lymphocytes after unrelated cord blood transplantation – a case report

**DOI:** 10.1111/j.1365-2257.2006.00809.x

**Published:** 2006-10

**Authors:** Y SHONO, T TOUBAI, S OTA, M IBATA, S MASHIKO, D HIRATE, Y MIURA, S UMEHARA, N TOYOSHIMA, J TANAKA, M ASAKA, M IMAMURA

**Affiliations:** * Department of Hematology and Oncology, Hokkaido University Graduate School of Medicine Kita-ku, Sapporo, Japan; † Department of Gastroenterology and Hematology, Hokkaido University Graduate School of Medicine Kita-ku, Sapporo, Japan

**Keywords:** Cord blood transplantation (UBT), naïve B lymphocyte, CD19^+^27^−^IgD^+^ B cells, IL-6, immunological reconstitution

## Abstract

A 33-year-old woman underwent unrelated cord blood transplantation (U-CBT) for myelodysplastic syndrome (MDS)-related secondary AML. She showed impressive increases in the number of CD19^+^ B cells in bone marrow and CD19^+^27^−^IgD^+^ B cells in peripheral blood from about 1 month to 3 months after U-CBT. The serum level of IL-6 temporarily increased after transplantation, and this increase seemed to be correlated with the expansion of CD19^+^ B cells. Although, compared with BMT, little is known about the kinetics of hematological and immunological reconstitution in U-CBT, there was initial B-cell recovery after CBT as some described. This B cell recovery may be associated with a high number of B-cell precursors present in cord blood (CB). The phenomenon of naïve B lymphocyte expansion that we found might be associated with a high number of B-cell precursors present in CB.

## Case history

**Figure 1:**
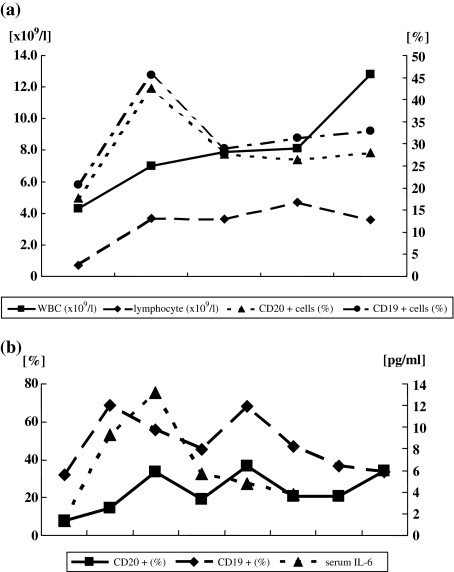
Levels of WBCs, peripheral lymphocytes, peripheral CD19^+^ lymphocytes, and CD20^+^ lymphocytes after U-CBT (a) and levels of CD19^+^ lymphocytes and CD20^+^ lymphocytes in BM and serum IL-6 after U-CBT (b). CD19^+^ and CD20^+^ lymphocyte counts peaked on day 63.

**Figure 2:**
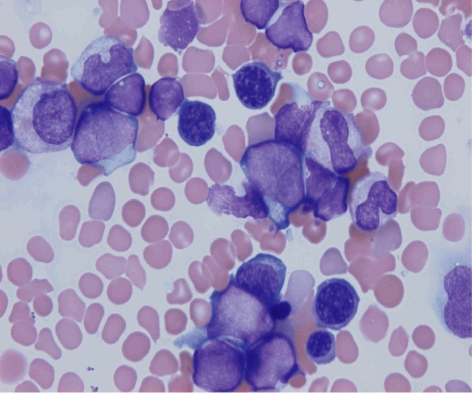
Bone marrow smear. (May-Gimza stain ×1000). Bone marrow aspirate on day 62 shows various types of blastic-appearing lymphocytes with a high nuclear/cytoplasmic ratio and a fine nucleoreticulum. These lymphocytes also had markedly reduced granulation.

**Figure 3:**
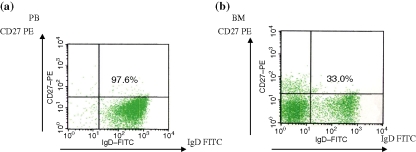
Phenotype analysis of PB (a) and BM (b) B lymphocytes. Three B-cell subsets were separated according to surface IgD and CD27 expression on CD19^+^ B cells. Although >95% of CD19^+^ B cells in PB showed IgD^+^CD27^−^ naïve B cell type, we could not detect them in BM.

A 33-year-old woman with myelodysplastic syndrome (MDS)-related secondary acute myelogenous leukemia (AML) underwent unrelated cord blood transplantation (U-CBT) from HLA 1-antigen (DRB1) mismatched donor in April 2003. The conditioning regimen consisted of total body irradiation (TBI, 12 Gy) on days −9, −8, and −7, and cytarabine (Ara-C) at 3 g/m^2^ every 12 h on days −6 and −5, and cyclophosphamide (CY) at 60 mg/kg once daily on days −4 and −3. FK 506 and short-term methotrexate (MTX) were used for graft-versus-host disease (GVHD) prophylaxis. The number of infused nucleated cells and CD34^+^ cells were 3.0 × 10^7^/kg and 2.8 × 10^4^/kg respectively. Neutrophil and platelet engraftment was achieved on day 18 and day 28 respectively. We examined donor-type chimerism in the peripheral blood (PB) mononuclear cells by the procedure reported in detail previously (Tsutsumi *et al.*, 2002), complete donor type revealed on day 28. Acute GVHD was observed about 2 weeks after transplantation (skin, stage 2; gut, stage 3; overall, grade III), but the symptoms gradually subsided after prednisolone administration at a single dose of 1 mg/kg. FK506 therapy was stopped on day 31 when GVHD symptoms had disappeared. The patient showed an impressive increase in the number of CD19^+^ B cells in bone marrow (BM) and PB starting approximately 1 month after U-CBT (Figure 1a,b). Bone marrow aspirate on day 62 showed various types of blastic-appearing lymphocytes (47.4%) with a high nuclear/cytoplasmic ratio and a fine nucleoreticulum (Figure 2). These lymphocytes also had markedly reduced granulation. These lymphocytes also expressed CD20 antigen (data unshown). This increase was not apparently related to a defective control of Epstein–Barr virus (EBV) infection or reactivation, which was confirmed by the negative results of PCR and Southern blot analysis on day 62. EBV serological examination before U-CBT revealed viral capsid antigen (VCA)-IgG 40×, VCA-IgM < 10×, VCA-IgA < 10×, early antigen for DR components (EADR)-IgG < 10×, EADR-IgA < 10× and EBV nuclear antigen (EBNA) 10×. Moreover, EBV serological examination on day 62 after U-CBT revealed VCA-IgG 160×, VCA-IgM < 10×, VCA-IgA < 10×, EADR-IgG < 10×, EADR-IgA < 10× and EBNA 40×. The patient remained afebrile, and there was no newly emerged lymphadenopathy. Cytomegalo virus (CMV) antigenemia was negative at all times after the transplantation, and there was no indication of any bacterial infection. Results of serological examinations of Parvovirus B19 and Herpes simplex virus (HSV) were negative, and Human papiloma virus- 6 (HHV-6) was not examined. Immunoglobulin heavy chain (IgH) clonality analysis showed negative rearrangement. To evaluate the correlation between immunological reconstitution and circulating serum cytokines, serum levels of interleukin-2 (IL-2), interleukin-6 (IL-6), interleukin-10 (IL-10) and interferon-*γ* (IFN-*γ*) were also examined. We found that the serum level of IL-6 temporarily increased after transplantation and that this increase correlated with the expansion of CD19^+^ B cells (Figure 1b). However, IL-2, IL-10 and IFN-*γ* were not detected. The CD19^+^ B cells were analyzed in detail using anti CD27 Ab and anti IgD (Figure 3a,b). IgD^+^ CD27^−^ cells (naïve B cells) in the CD19^+^ B cells increased in BM and PB. Results of other routine examinations were unremarkable except for the presence a relatively high percentage of lymphocytes in PB. Sequential analysis of chimerism status of CD19^+^cells was performed, and complete donor chimerism persisted after transplantation. Taking all of these facts into consideration, we regarded this phenomenon as a nonmalignant state that resulted from a peculiar immunologic reconstitution after U-CBT, and we just followed up the patient carefully. The number of CD19^+^ B cells in BM and PB gradually normalized afterwards. Further, we observed that CD4^+^CD8^+^ cell ratios in PB were 0.42, 1.17 and 1.29 at 1, 3 and 4 months respectively. Absolute counts of CD3^+^ cells, CD4^+^ cells and CD8^+^ cells of >500/*μ*l were achieved at 3, 4 and 4 months respectively. Immune reconstitution in these lymphocyte subsets was earlier than that reported by Giraud *et al.* (2000).

## Discussion

An increase in lymphocytes in PB approximately 2 months after U-CBT has been described in a few reports (Locatelli *et al.*, 1996; Elhasid *et al.*, 2000; Moretta *et al.*, 2001) but there was no mention of lymphocytes in BM in these reports. It has been reported that CD19^+^ B cells showed prompt recovery after CBT (Giraud *et al*., 2000, Niehues *et al.*, 2001; Inoue *et al.*, 2003). Giraud *et al*. (2000) found that B cells the recovered early and at 6 and 9 months they constituted the predominant lymphocyte subset. They also found that the absolute CD19^+^ B cell number was elevated at 12 months in all of their child patients except for the one with severe chronic GVHD after unrelated CBT. Inoue *et al.* (2003) compared immune reconstitution after allogeneic CBT and CD34^+^ stem cell transplantation (CD34^−^ SCT) with that after BMT in children and found that both the number and percentage of CD19^+^ B cells were higher after CBT. Moreover, B cell recovery may be associated with a high number of B-cell precursors present in CB (Arakawa-Hoyt *et al.*, 1999; Knutsen & Wall, 2000). However, there were no data of subpopulations of B cells after CBT. We analyzed the B lymphocytes of the adult patient after U-CBT using three subpopulations on the basis CD27 and IgD expression. Klein, Rajewsky and Kuppers (1998) reported that PB of healthy donors showed considerable variation in the percentages of the B-cell subsets, with 29–65% IgD^+^CD27^−^, 6.5–22% IgD^+^CD27^+^ and 13–43% IgD^−^CD27^+^ B cells. Although the percentages of B-cell subsets in BM were unclear, our case showed a clear increase in naïve B cells. It has been hypothesized that this striking expansion of B cells is due to the absence of long-term memory B cells; long-term memory B cells are usually found in adult BM, whereas only immature naïve B lymphocytes are found in cord blood (Locatelli *et al.*, 1996). It is conceivable that neonatal B lymphocytes or their precursors present in cord blood maintain an efficient self-renewal capacity even after being transplanted into an allogeneic host.

We studied serum levels of IL-2, IL-6, IL-10 and IFN-*γ* to examine the relationship between immunological reconstitution and circulating serum cytokines. IL-6 was the only cytokine that increased after U-CBT, and its increase was correlated with the expansion of CD19^+^ B cells (Figure 2-b). In regard to the increases of serum IL-6 level in allogeneic BMT, this phenomenon was observed early after the transplantation and appeared to be one of the causative cytokines for GVHD induction (Imamura *et al.*, 1994). We cannot demonstrate a clear cause-and-effect relationship between the increases in serum level of IL-6 and number of CD19^+^ B cells, however, this correlation is interesting and further studies are expected.

In conclusion, an impressive increase in CD19^+^ B cells, especially naïve B cells, in BM and PB starting about 1 month after U-CBT was found, and this phenomenon of naïve B lymphocyte expansion might be associated with a high number of B-cell precursors present in CB.

## References

[b1] Arakawa-Hoyt J, Dao MA, Thiemann F, Hao QL, Ertl DC, Weinberg KI, Crooks GM, Nolta JA (1999). The number and generative capacity of human B lymphocyte progenitors, measured *in vitro* and *in vivo*, is higher in umbilical cord blood than in adult or pediatric bone marrow. Bone Marrow Transplantation.

[b2] Elhasid R, Ben Arush MW, Pollack S, Tavor K, Streichman S, Postovsky S, Haddad N, Rowe JM (2000). Immune and hematopoietic reconstitution after transplantation of cord blood progenitor cells: case report and review of the literature. Leukemia.

[b3] Giraud P, Thuret I, Reviron D, Chambost H, Brunet C, Novakovitch G, Farnarier C, Michel G (2000). Immune reconstitution and outcome after unrelated cord blood transplantation: a single paediatric institution experience. Bone Marrow Transplantation.

[b4] Imamura M, Hashino S, Kobayashi H, Kubayashi S, Hirano S, Minagawa T, Tanaka J, Fujii Y, Kobayashi M, Kasai M, Sakurada K, Miyazaki T (1994). Serum cytokine levels in bone marrow transplantation: synergistic interaction of interleukin-6, interferon-gamma, and tumor necrosis factor-alpha in graft-versus-host disease. Bone Marrow Transplantation.

[b5] Inoue H, Yasuda Y, Hattori K, Shimizu T, Matsumoto M, Yabe M, Yabe H, Tsuchida F, Tanaka Y, Hosoi G, Sako M, Kato S (2003). The kinetics of immune reconstitution after cord blood transplantation and selected CD34^+^ stem cell transplantation in children: comparison with bone marrow transplantation. International Journal of Hematology.

[b6] Klein U, Rajewsky K, Kuppers R (1998). Human immunoglobulin (Ig)M^+^IgD^+^ peripheral blood B cells expressing the CD27 cell surface antigen carry somatically mutated variable region genes: CD27 as a general marker for smatically mutated (memory) B cells. Journal of Experimental Medicine.

[b7] Knutsen AP, Wall DA (2000). Umbilical cord blood transplantation in severe T-cell Immunodeficiency disorders: Two-year experience. Journal of Clinical Immunology.

[b8] Locatelli F, Maccario R, Comoli P, Bertolini F, Giorgiani G, Montagna D, Bonetti F, De Stefano P, Rondini G, Sirchia G, Severi F (1996). Hematopoietic and immune recovery after transplantation of cord blood progenitor cells in children. Bone Marrow Transplantation.

[b9] Moretta A, Maccario R, Fagioli F, Giraldi E, Busca A, Montagna D, Miniero R, Comoli P, Giorgiani G, Zecca M, Pagani S, Locatelli F (2001). Analysis of immune reconstitution in children undergoing cord blood transplantation. Experimental Hematology.

[b10] Niehues T, Rocha V, Filipovich AH, Chan KW, Porcher R, Michel G, Ortega JJ, Wernet P, Gobel U, Gluckman E, Locatelli F (2001). Factors affecting lymphocyte subset reconstitution after either related or unrelated cord blood transplantation in children-a Eurocord analysis. British Journal of Haematology.

[b11] Tsutsumi Y, Tanaka J, Kato N, Zhang L, Mori A, Kobayasi R, Kasai M, Asaka M, Imamura M (2002). Analysis of mixed chimerism in patients after allogeneic stem cell transplantation using a capillary electrophoresis system. Acta Haematologica.

